# Supplementary material for: Native dynamics and allosteric responses in PTP1B probed by high‐resolution HDX‐MS
Source: Protein Sci. 2024 May 27;33(6):e5024. doi: 10.1002/pro.5024 (PMC11129624; doi:10.1002/pro.5024)
Supplement: Supplementary file 1 — Appendix S1. Supporting information. [file PRO-33-e5024-s001.pdf]

Supplemental Information

Native dynamics and allosteric responses in PTP1B probed by high-resolution HDX-MS

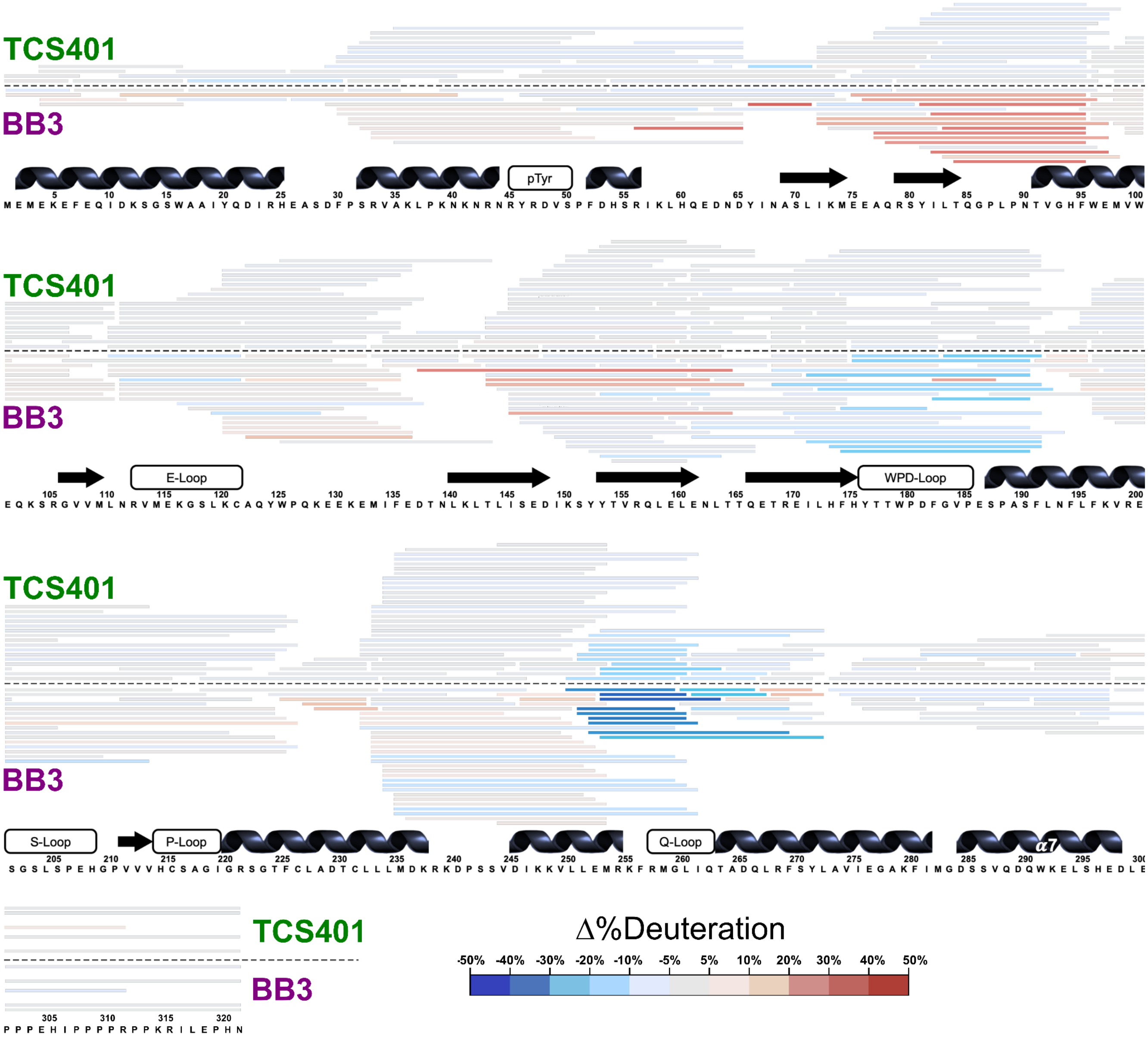

Figure S1: Difference HDX map for TCS401 minus apo PTP1B at 30 seconds.
Peptide maps for the TCS401 and BB3 conditions, with peptides colored based on the difference in HDX rate between liganded minus apo conditions at 30 seconds of labeling time.

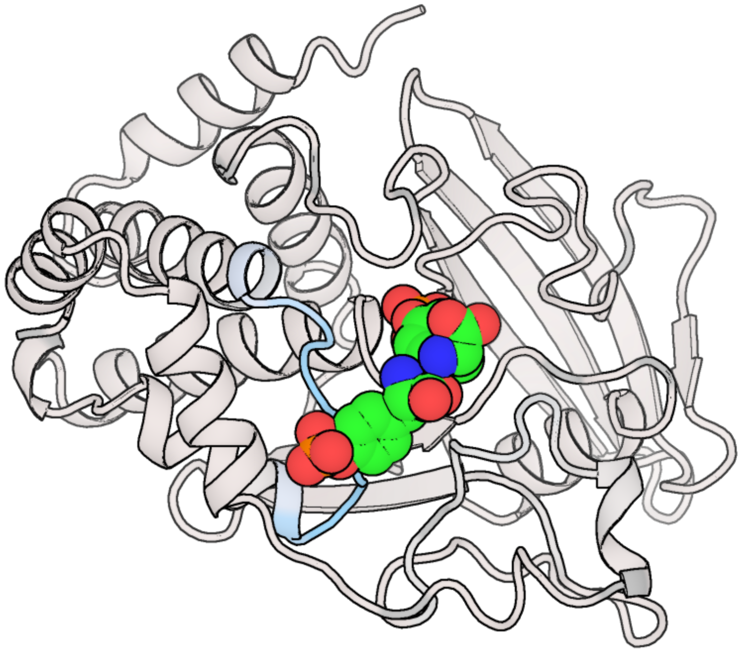

Figure S2: Structural view of TCS401 HDX differences in the context of two pTyr sites.
Same as main **Fig. 5A** (30 seconds labeling) but with TCS401 omitted and the two pTyr residues from PDB ID 1PTY overlaid (green).

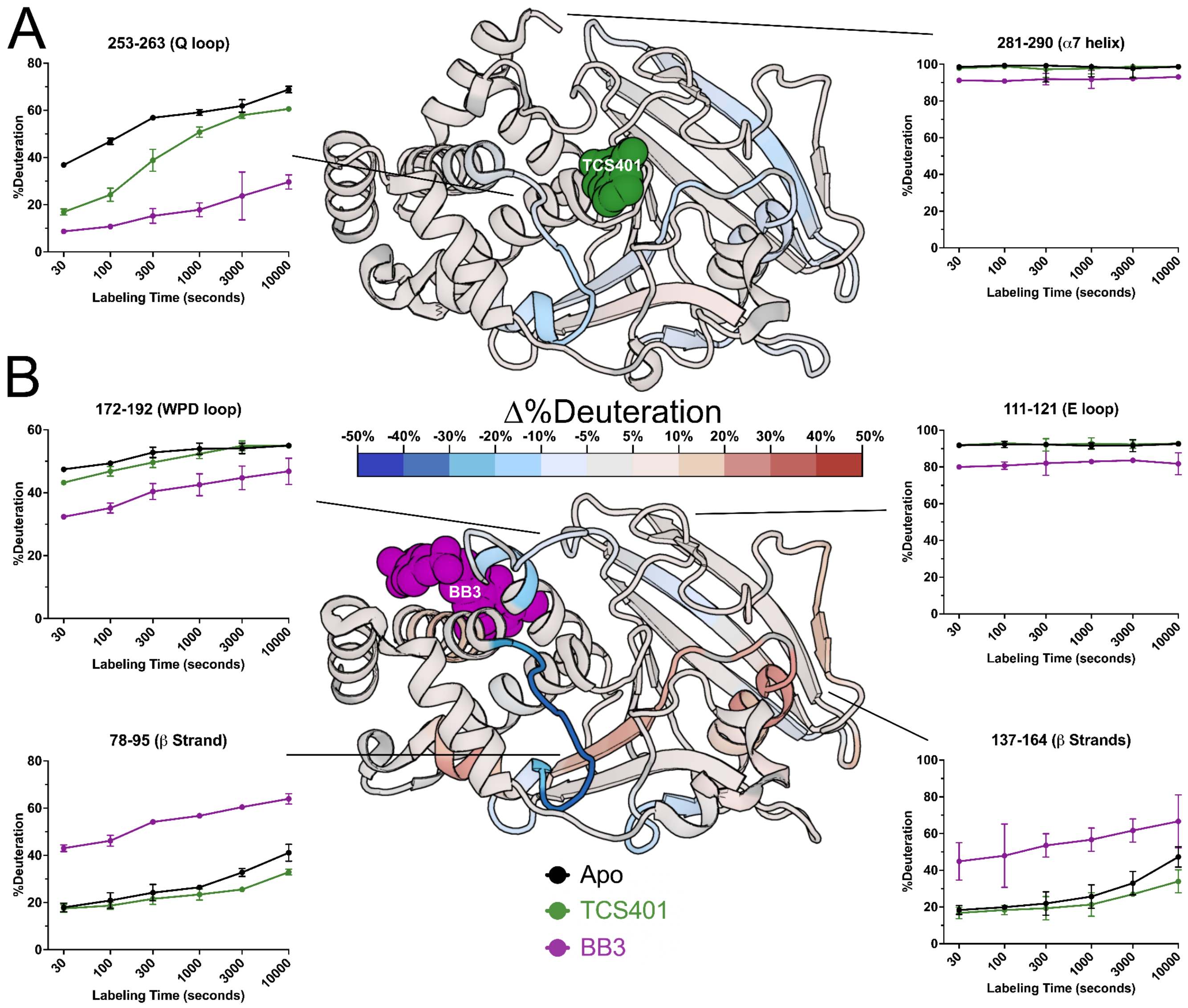

Figure S3: Effects of active-site inhibitor (TCS401) and allosteric-site inhibitor (BB3) after extended labeling time.

Same as **Fig. 5** but with coloring of residues at 10,000 seconds of labeling time.

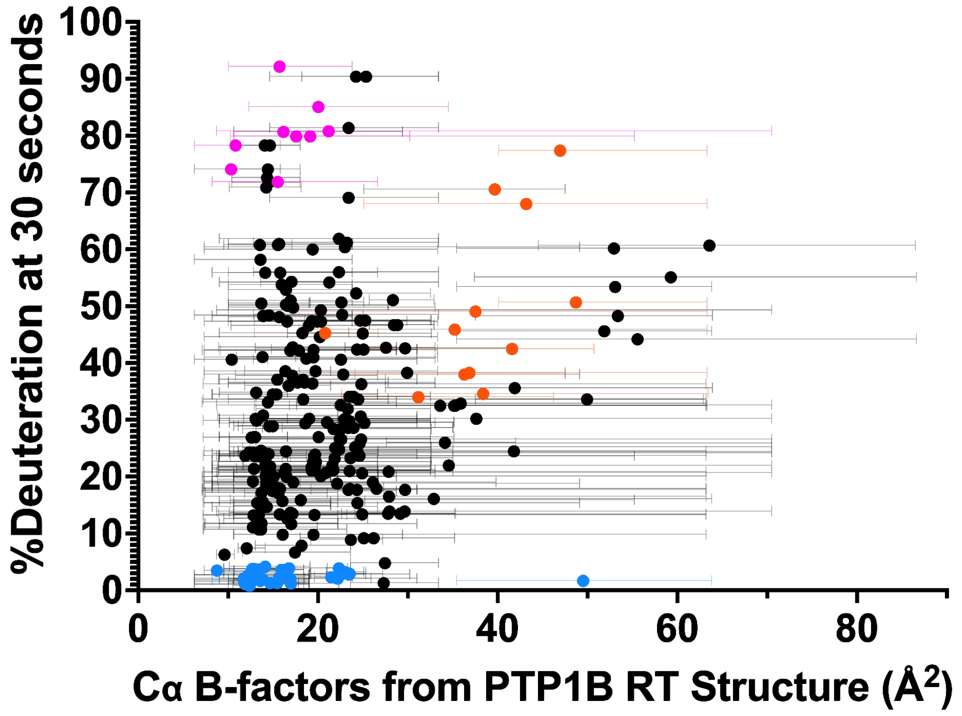

Figure S4: Room-temperature B-factors correlated with HDX-MS deuteration levels.
Same as **Fig. 3B**, but using Cα B-factors from a representative room-temperature crystal structure of PTP1B (PDB ID 6B8X). Peptides colored blue, magenta, and orange correspond to the same colored peptides in **Fig. 3B-E**. B-factors are occupancy-weighted for residues with alternate conformations.

Data S1: Fractional deuterium uptake for all three conditions.
Spreadsheet containing HDX and pseudo-ensemble data.
Tab 1: Peptide raw centroids for all three protein conditions across all time points.
Tab 2: Quantitative HDX data for apo PTP1B at 30 seconds of labeling time.
Tab 3: Cα RMSFs from pseudo-ensemble of PTP1B structures.
